# Criterion validity of the ActiGraph and activPAL in classifying posture and motion in office-based workers: A cross-sectional laboratory study

**DOI:** 10.1371/journal.pone.0252659

**Published:** 2021-06-02

**Authors:** Thomas Radtke, Manuel Rodriguez, Julia Braun, Holger Dressel

**Affiliations:** 1 Division of Occupational and Environmental Medicine, Epidemiology, Biostatistics and Prevention Institute, University of Zurich and University Hospital Zurich, Zurich, Switzerland; 2 Epidemiology, Biostatistics and Prevention Institute, University of Zurich, Zurich, Switzerland; University of Ottawa Heart Institute, CANADA

## Abstract

**Background:**

The ActiGraph and activPAL monitors are the most frequently used thigh-worn devices to measure motion and posture, but the criterion validity to measure sitting, standing and postural transfer in the office setting is not known. *Research question*: To examine the criterion validity of the ActiGraph and activPAL activity monitors in repeatedly measuring a variety of different postures and motion in the office setting.

**Methods:**

Twenty office workers from the University of Zurich wore an ActiGraph and activPAL during two identical laboratory experiments lasting approximately 60 minutes each, within a maximum of 7 days. The experimental setting consisted of a standard computer office workstation with an electrically powered height-adjustable desk, a swivel chair without arm rests, a standard chair, a footrest, and a bookcase. The protocol consisted of 24 pre-defined tasks mimicking sitting, standing, stepping, and postural transitions around the workplace. All tasks were supervised and observed by the same experimenter.

**Results:**

In repeated measurements (40 individual experiments), the percentages of correctly classified tasks for the ActiGraph and activPAL were, respectively, 100% vs. 85% for sitting, 87% vs. 100% for standing, and 100% vs. 73% for postural transitions. Both monitors correctly identified all stepping tasks. The activPAL misclassified sitting with legs outstretched, and sitting with both feet placed beneath the chair, as standing ~25–70% and 45% of the time, respectively. The ActiGraph misclassified standing with the right foot on a footrest as sitting in 65% of events.

**Conclusions:**

The ActiGraph appears to be slightly more sensitive than the activPAL with respect to the measurement of sitting and postural transitions of short duration, whereas the activPAL seems to be slightly more accurate in capturing standing postures. This knowledge will help guide researchers to choose the best suitable monitor for their research setting.

## 1. Introduction

Insufficient physical activity and prolonged sitting are a major public health concern [1] and associated with increased all-cause and cardiovascular disease risk mortality [2–4]. Targeting insufficient physical activity is one of the global priorities of the World Health Organization, aimed to prevent and treat non-communicable diseases [5]. Office workers spend a large proportion of their work time sedentary [6] and are therefore vulnerable to increased risk for deleterious health outcomes and a target group for interventional research. To date, very low- to low-quality evidence exists for the effects of workplace interventions (e.g., sit-stand desk, policy changes, provision of information, and counseling) to reduce sedentary time (sitting) at work [7]. Some of the major challenges of human sedentary behavior research are confusion about its definition and conceptualization and a lack of standardized measurement methodology [8]. Sedentary behavior is a complex concept and defined as any waking behavior characterized by low energy expenditure (≤1.5 metabolic equivalents) while in a sitting, reclining, or lying posture [8]. While self-reported assessment of workplace sitting time and breaks from sitting shows fair criterion validity in comparison with activity monitors, substantial under- and/or overestimation limits its use on an individual level [9, 10]. Technical advances in activity monitor sensors allow sedentary behavior and body posture to be quantified in the laboratory setting, at the workplace, and during daily life activities [9, 11–14]. Two frequently used thigh-worn activity monitors that use an inclinometer function to measure sitting/lying, standing, stepping, and postural transitions are the ActiGraph and activPAL^TM^ (hereafter referred to as activPAL) [6, 15]. The two monitors have been compared in laboratory and field research and differ in their accuracy in classifying postures and motion, likely because of their different data processing algorithms [11, 13, 16, 17]. To our knowledge, no previous study has compared the validity of the ActiGraph and activPAL in the detection of typical body postures and motion in the workplace. We therefore designed a laboratory-based experimental study to assess the criterion validity of the activPAL and ActiGraph in comparison with direct observation, measuring a variety of different postures and motions in the office setting to test the capacity limits of each of the devices. Furthermore, to assess the reproducibility of both monitors to accurately measure postures and motion at the workplace, we repeated the experiment following a strictly standardized testing protocol.

## 2. Materials and methods

### 2.1 Study sample

Twenty-two subjects (13 female) with a mean ± SD age of 38 ± 11 years and a body mass index of 23 ± 3 kg•m^-2^ employed at the Epidemiology, Biostatistics, and Prevention Institute (EBPI) of the University of Zurich participated. All participants confirmed not suffering from any health-related problems limiting postural movements in the office and reported not having an allergy to adhesive tape.

### 2.2 Ethics statement

This study does not fall under the scope of the Human Research Act. The Ethical Committee of the Canton of Zurich confirmed with a clarification of responsibility that ethical approval was not necessary for this study (2016–00464). We systematically obtained verbal consent from all study participants. Written informed consent (i.e., signature by study participant) is not mandatory for a project with minimal risks. The investigators contacted potentially eligible subjects at the EBPI, explained the purpose of the project and recorded the verbal consent status prior to participating in the study.

### 2.3 Experimental setting

Study participants were invited to the laboratory facility at the EBPI to take part in two identical experiments lasting approximately 60 minutes each and a maximum of 7 days apart (3 ± 2 days). The experimental setting consisted of a standard computer office workstation with an electrically powered, height-adjustable desk (180 x 80 cm, Ergodata AG, Zurich, Switzerland), a swivel chair without arm rests (Campos, Interstuhl, Germany), a bookcase, and a footrest. In addition, a standard chair was used to simulate sitting positions during breaks or meetings. The office chair and height-adjustable desk were individually adjusted according to the current Swiss ergonomic recommendations of the Federal Coordination Commission for Occupational Safety [18].

### 2.4 Experimental tasks

The protocol consisted of 24 pre-defined tasks representing sitting (N = 11), standing (N = 5), stepping (N = 5), and postural transitions (N = 3) around the workplace, with each task lasting a maximum of 2 minutes. The detailed experimental protocol including the description of the different tasks can be found elsewhere (S1 Appendix). At the end of the experiments, the participants were asked open questions about positions that the investigators missed to address compared to their personal habits and regarding the wearing comfort of the two activity monitors.

### 2.5 Additional experimental measurements

**Fig 1:**
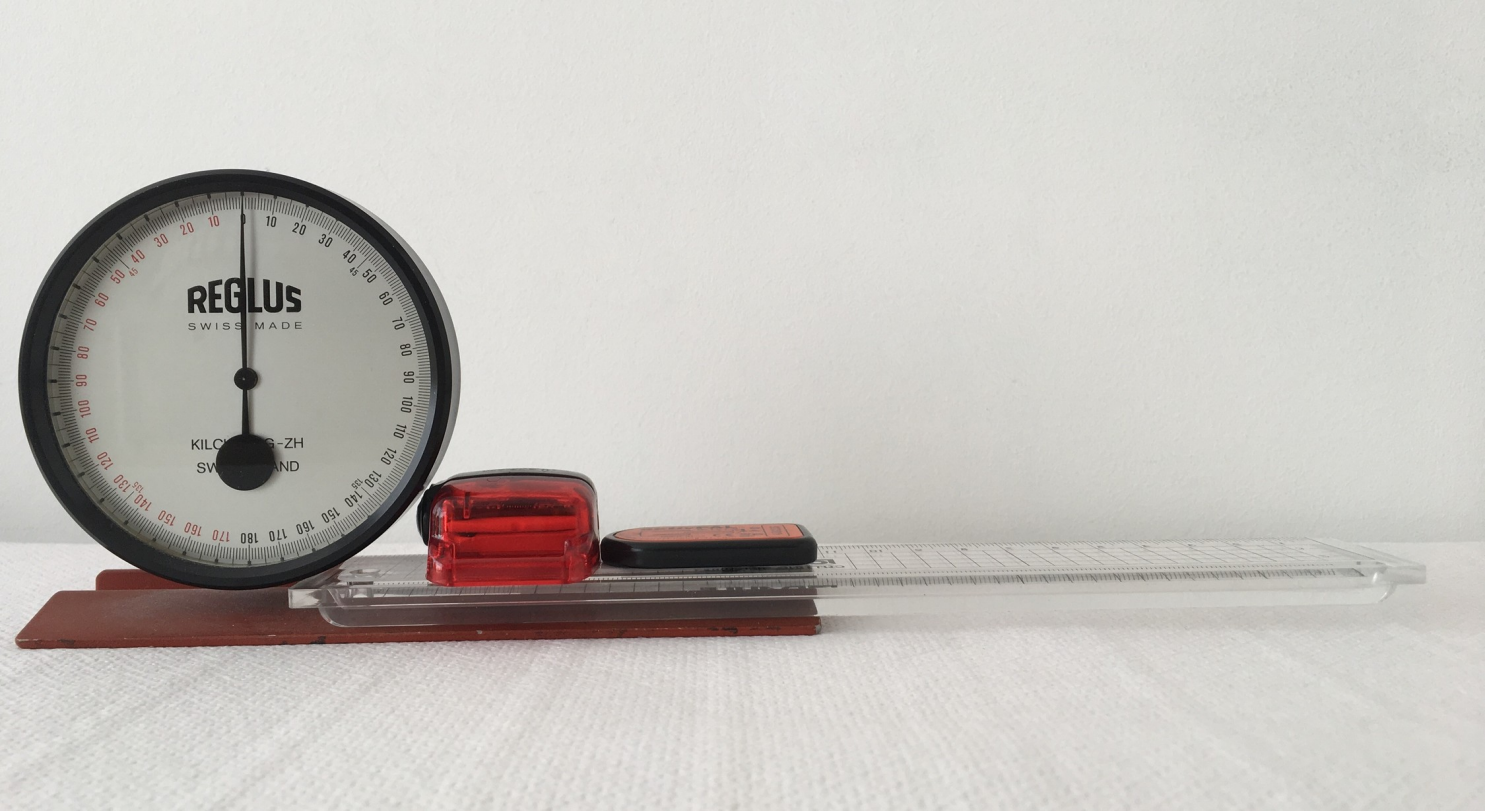
Experimental setup for protractor measurements.

Based on previous work showing that the activPAL misclassifies sitting with outstretched legs as standing, 14% of the time [11], we performed additional measurements in which the activity monitors were attached to a protractor (Reglus, Kilchberg, Zurich, Switzerland) to measure the output of the devices when positioned at different angles (Fig 1). In brief, the devices were moved in 10° steps covering thigh positions representing a headstand, sitting, and standing (headstand = 90° on the right protractor scale; sitting = 0° on the protractor scale (Fig 1), and standing = 90° on the left protractor scale). At each 10° angle, the examiner held the protractor in his hand for two minutes. The same exercise was repeated, stepping the movement of the protractor in the opposite direction.

### 2.6 Activity monitors

The activPAL micro (PAL Technologies Ltd, Glasgow, Scotland, UK) is a small, lightweight, thigh-worn accelerometer/inclinometer that has been designed to quantify periods of an individual`s sedentary and upright positions and daily activities using proprietary algorithms [2–4]. The device measures acceleration at a sampling frequency of 20 Hz (80 Hz can be used in research mode). It uses information from static acceleration and the angle of the thigh to classify postures (lying/sitting vs. upright), and dynamic acceleration (due to body movement) to determine stepping. The activPAL has been considered to be an accurate and precise monitor to assess sedentary behavior in various populations [5–7].

The ActiGraph wGT3X-BT is a small and lightweight triaxial accelerometer (ActiGraph LLC, Pensacola, Florida, USA), and the most commonly used device in physical activity research and large-scale epidemiological studies [8]. The ActiGraph measures acceleration with a sampling frequency between 30–100 Hz and has an inclinometer algorithm to detect sitting and standing postures.

Both devices were placed at the anterior aspect of the right thigh, midway between the hip and knee joint [11]. The activPAL was fixed with double-sided adhesive tape and the ActiGraph was fixed with its commercially available elastic belt.

### 2.7 Direct observation

We used direct observation as the validation criterion to determine the different tasks and postures to be evaluated in the experimental protocol (S1 Appendix). All laboratory experiments were supervised by the same observer (MR) throughout the study. The observer explained all tasks, placed and controlled the correct position of the activity monitors, and provided detailed instructions during the experiments.

### 2.8 Data preparation and processing

The activity monitors were synchronized with the system clock of the computer and initialized with their respective manufacturer’s software. The activPAL was programmed to record data at a sampling frequency of 20 Hz (activPAL^TM^ Research Edition version 7.2.32), and the minimum sitting/upright period to detect a new posture was set at 1 second to be able to capture rapid transitions between different postures. The ActiGraph was initialized to collect raw data at a sampling frequency of 30 Hz (Actilife software version 6.13.3). After completion of each individual experiment, the raw data were downloaded at a sampling epoch of 15 seconds and saved to a csv spreadsheet (Microsoft Excel 2018, Version 16.16.8) for further data analysis. The following data from both the activPAL and ActiGraph were considered for further analysis: time, upright or standing time, stepping time, and sedentary or sitting/lying time. The beginning of each individual task (coded as sitting, standing, or stepping) was identified by the annotated time. The displayed result from the monitor was compared with the requested position defined in the experimental protocol, in order to determine whether the task was correctly identified by the device or not. The authors did not extract additional raw data from the devices. Details of the evaluation criteria that had to be fulfilled to label a task as “correctly coded” are given in the S1 Table.

### 2.9 Data analyses and sample size considerations

A priori, we defined to include a convenience sample of 20 participants, similar to a previous study comparing activPAL and ActiGraph under laboratory and free-living conditions [11], and planned to replace drop-outs. We calculated the numbers and percentages of tasks correctly coded by each monitor, with the reference being direct observation. Additionally, we clustered all similar tasks into groups: i) sitting (N = 11), ii) standing (N = 5), iii) stepping (N = 5), and iv) postural transition (N = 3), and also calculated the percentage of correctly coded tasks. In addition, we tested one special position, with the person standing with the right foot placed on a bookcase (~ 90° hip angle), that we expected to be classified as sitting by both monitors. The classification accuracy of each individual task to determine the correct posture between the two time points was analyzed with the McNemar test, a test for matched-pairs binary data. Statistical analyses were performed using the statistical software package SPSS version 23 (IBM Corp. Armont, NY, USA).

## 3. Results

Of 22 participants, 2 had to be excluded because the maximum allowed time difference of 7 days between the first and second study visit, per the study protocol, could not be met. Therefore, 20 participants were included in the final analysis. During one individual experiment, the activPAL malfunctioned, classifying the first eight tasks incorrectly as standing and thus resulting in slightly lower agreement of repeated measurements (Table 1). Of note, the activPAL was 100% accurate for the given tasks, when the malfunctioning device was excluded from the analysis (Table 1).

**Table 1:** Number (%) of tasks correctly classified by the activPAL and ActiGraph in repeated measurements.

	activPAL^TM^ micro	ActiGraph wGT3X-BT
ASSIGNED TASKS	Number (%) of correctly classified tasks per person in the 1^st^ *and* 2^nd^ experiments	Number (%) of correctly classified tasks per person in the 1^st^ *and* 2^nd^ experiments
**SITTING (N = 11)**		
Typing text on keyboard	19 (95)*	20 (100)
Feet placed on footstool	20 (100)	20 (100)
Moving backrest forward and backward	19 (95)*	20 (100)
Fidgeting with the right leg	19 (95)*	20 (100)
Right leg crossed at the left knee	20 (100)	20 (100)
Feet placed on the desk	20 (100)	20 (100)
Backrest fixed fully backward	19 (95)*	20 (100)
Legs outstretched	6 (30)	20 (100)
Legs outstretched & backrest fully backward	15 (75)	20 (100)
On a chair with both feet on the ground	18 (90)	20 (100)
On a chair with lower legs bent far back under the seat of the chair	11 (55)	20 (100)
**STANDING (N = 5)**		
In front of the pin board	20 (100)	20 (100)
Desk adjusted for standing position	20 (100)	20 (100)
Desk adjusted for standing position & typing text on keyboard	20 (100)	20 (100)
Waiting for printout in front of the printer	20 (100)	20 (100)
Desk adjusted for standing position & right foot placed on footrest	20 (100)	7 (35)
**STEPPING (N = 5)**		
Walking outside around the building	20 (100)	20 (100)
Stairs, ascending (two flights)	20 (100)	20 (100)
Stairs, ascending (one flight)	20 (100)	20 (100)
Stairs, descending (three flights)	20 (100)	20 (100)
Stairs, descending (one flight)	20 (100)	20 (100)
**POSTURAL TRANSFER (N = 3)**		
Getting up from and sitting down on a chair to open and close the window *(Sit–Step–Stand–Step–Sit–Step–Stand–Step–Sit)*	18 (90)	20 (100)
Getting up from a chair and walking a few meters to take a clipboard from the bottom of a bookcase, returning to the chair and sitting down again *(Sit–Step–Squat–Step–Sit)*	19 (95)*	20 (100)
Standing in front of the printer, taking the printout, walking a few meters and squatting down to place the printout in the lower part of the bookcase *(Stand–Step–Squat–Stand–Step)*	7 (35)	20 (100)
**SPECIAL POSITION (N = 1)**		
Standing with the right foot placed on the bookcase with ~90° hip angle	18 (90)	20 (100)

Number (%) of correctly classified tasks for each activity monitor based on repeated individual measurements (N = 40) from 20 study participants. There were no differences in classification accuracy in detecting the pre-specified tasks in repeated measurements for either the activPAL or ActiGraph based on McNemar’s test statistics (S1 and S2 Tables).

*During one individual experiment, the activPAL monitor malfunctioned, coding the first tasks constantly as standing.

**Fig 2:**
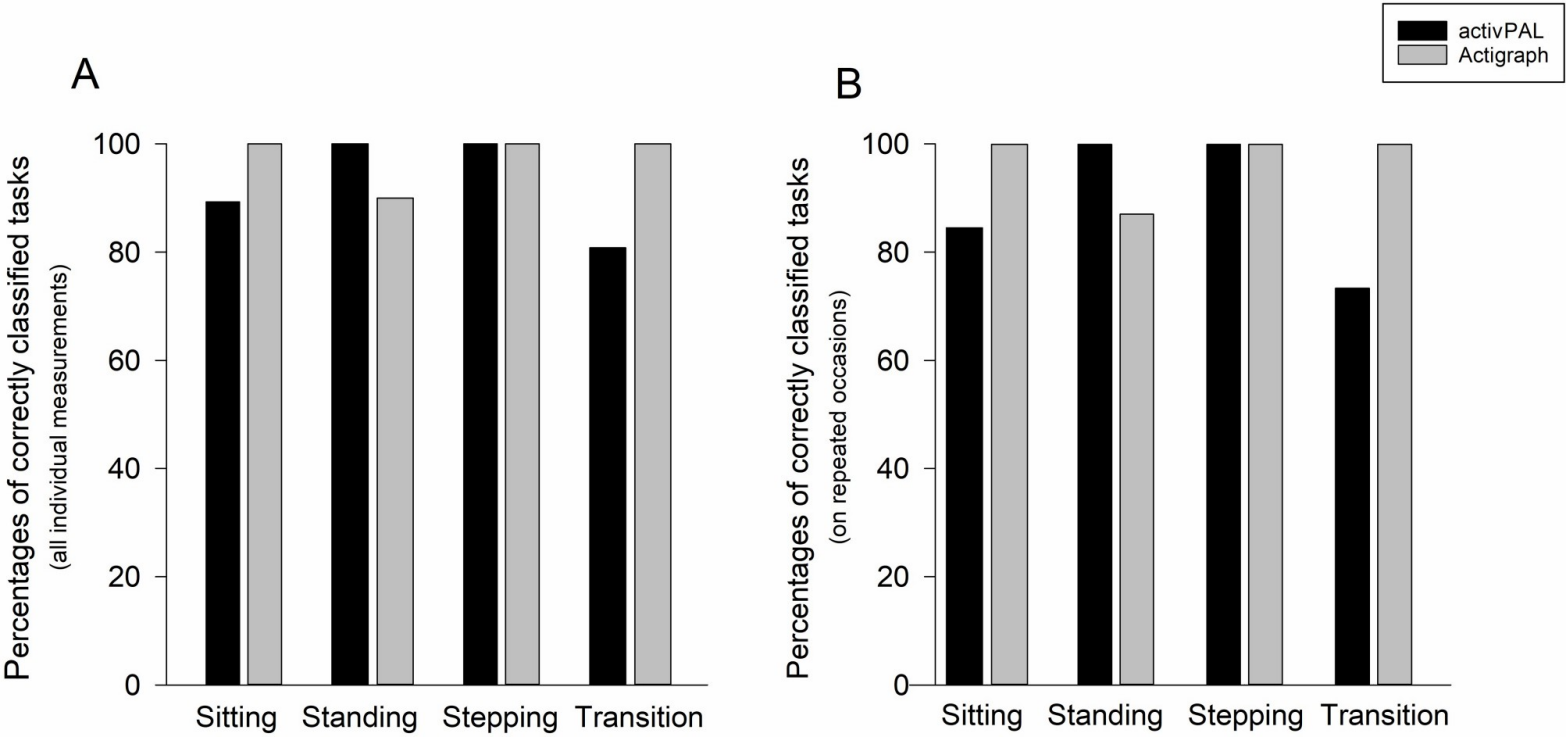
Overview of correctly classified tasks. Percentages of correctly classified tasks for the activPAL and ActiGraph considering all individual measurements (2A) and percentages of correctly classified tasks during both experimental visits, i.e., on repeated occasions (2B). Individual tasks were clustered into sitting (N = 11), standing (N = 5), stepping (N = 5) and postural transitions (N = 3).

The number (percentage) of correctly classified tasks per study participant during both experimental conditions is given in Table 1. There were no significant differences in the detection accuracy of workspace–related tasks for either activPAL or ActiGraph in repeated measurements (Tables 1 and S2 and S3). Fig 2 displays the percentages of correctly classified tasks categorized into sitting, standing, stepping, and postural transitions for activPAL and ActiGraph, considering all 40 individual measurements (Fig 2A), and the percentages of correctly classified tasks on repeated occasions during both experimental visits (Fig 2B). Detailed overviews of the classification accuracy of activPAL and ActiGraph for each individual task in repeated measurements are provided in the S2 and S3 Tables, respectively. All individual data are made available to reproduce the study results (S2 Appendix).

### 3.1 Sitting tasks

The ActiGraph monitor correctly classified all 11 sitting tasks on both occasions, whereas the activPAL correctly classified 3 of the 11 tasks correctly. The activPAL showed the greatest misclassification for sitting positions with the legs outstretched or with the lower legs bent far back under the seat of the chair (Table 1). In particular, there was a difference in the classification accuracy between sitting with legs outstretched with or without the backrest fully reclined (75% vs. 30% correctly classified).

### 3.2 Standing tasks

All five standing tasks were correctly classified by the activPAL and four of the five tasks were correctly classified by the ActiGraph. The ActiGraph misclassified standing with the right foot on the footrest as sitting in 65% of events (Table 1).

### 3.3 Stepping tasks

All stepping tasks were correctly classified by both activity monitors in repeated measurements (Table 1 and Fig 2).

### 3.4 Postural transfer

Postural transitions were all correctly classified by the ActiGraph monitor, whereas the activPAL misclassified each of the three postural transfer movements in 5, 10, and 65% of events (Table 1 and Fig 2).

### 3.5 Special position

The additional position, that we expected to be classified as sitting by both monitors, was correctly classified by the ActiGraph and activPAL in 100 and 90% of events in repeated measurements, respectively (Table 1).

### 3.6 Missing positions

The majority of participants confirmed that the office furniture and postures that we tested were typical of their everyday offices and work. The participants reported using alternative seating furniture in addition to the types we used in our experiment, such as an ergonomic chair, a stool, a seat ball, or seat wedge. One participant reported sitting cross-legged on the chair.

### 3.7 Protractor measurements

**Fig 3:**
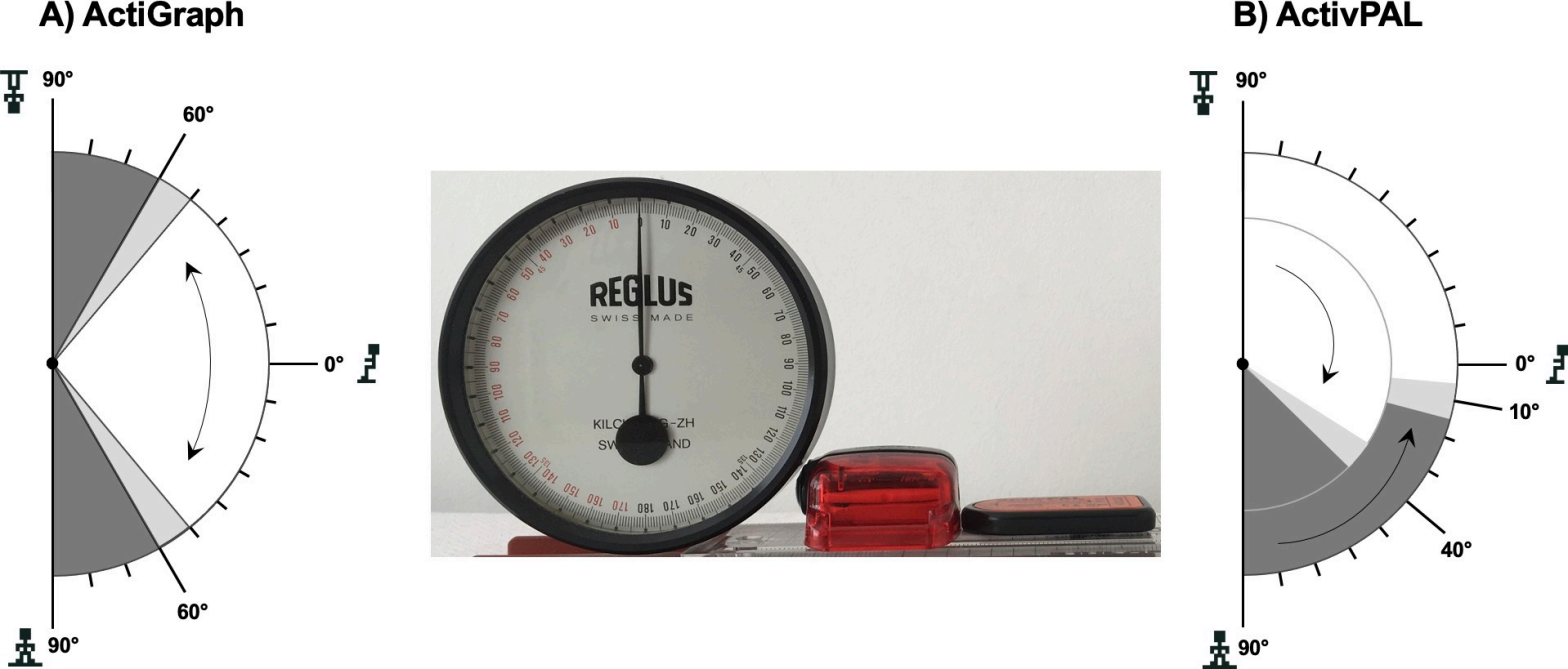
Graphical representation of the results of the angles measurements. Angle measurements for the ActiGraph (3A) and activPAL (3B) using a protractor. The picture in the middle shows the starting position with the devices placed parallel to the floor and the protractor scale reading 0°, representing sitting. The areas in white and grey represent the coded position and angle ranges relative to the horizontal. White = sitting, dark grey = standing, light grey = postural transition (sitting to standing or vice versa). The arrows show the direction in which the devices were moved. The double arrow in 3A shows that the coded position was independent from the direction in which the monitor was moved. The two arrows in 3B highlight differences in the coded posture and postural transition, depending in which direction the monitor was moved.

In repeated measurements with the protractor we found that ActiGraph classified as sitting any position at an angle between 0° and 50–60° from horizontal (0°), and as standing any position at an angle >50–60° from horizontal, regardless of the direction in which the monitor was moved (Fig 3A). In contrast to ActiGraph, we found different inflection points for the transition from sitting to standing (i.e., ~ 40° from horizontal) or standing to sitting (i.e., ~10° from horizontal) for activPAL (Fig 3B). ActivPAL, but not ActiGraph, categorized all activities as sitting—even when the monitor was moved into a headstand thigh position, in which a person would sit with strongly bent legs and the thighs close to the chest, knees pointing up.

### 3.8 Wearing comfort

Four participants (20%) found the activPAL to be more comfortable than the ActiGraph because they reported that the “thicker” device rubbed on their clothing. Five participants (25%) felt the elastic belt from the ActiGraph disturbing and one male participant felt it unpleasant to install the activPAL monitor on his hairy legs with the adhesive tape. Of note, discomfort maybe greater when the device is worn for longer periods. All other participants hardly noticed any discomfort while wearing the devices during the laboratory experiments.

## 4. Discussion

This study evaluated the criterion validity of the ActiGraph and activPAL in successfully determining postures and motion in desk-based office workers. Overall, the accuracies of both activity monitors in categorizing a variety of different postures and motion in the laboratory office setting were high, although some task-specific differences in non-standard postures were noted between monitors. In the workplace, the ActiGraph appears to be slightly more sensitive than the activPAL with respect to the measurement of sitting and postural transitions of short duration, whereas the activPAL seems to be slightly more accurate in capturing standing postures.

In general, both the ActiGraph and activPAL showed good accuracy for a large proportion of standard workplace-related tasks. With respect to non-standard tasks, the activPAL misclassified sitting with the legs outstretched, and sitting with the legs bent far back under the seat of the chair, as standing 70% and 45% of the time, respectively. Steeves et al. [11], using activPAL, also reported misclassification for sitting with legs outstretched and crossed at the ankle 14% of the time (70% in our study), when the ActiGraph was 100% accurate. In contrast, the Actigraph misclassified standing at the desk with the right foot placed on a footrest incorrectly as sitting, 65% of the time. The differences in classification accuracy between the two monitors result from their different algorithms and thigh angle specifications to classify postures as either sitting or standing [11]. Additional experiments with a protractor—an instrument used for measuring angles—showed substantially different angle thresholds for the ActiGraph and activPAL in classifying postures.

ActiGraph uses a complex hierarchical algorithm, including vector magnitude counts and information on units of gravity from the z-axis and x-axis, to differentiate between stepping, standing, and sitting/lying [11]. Details on units of gravity and angle thresholds for the different axes can be found elsewhere [11, 19]. The activPAL thigh-worn device determines posture on the basis of thigh inclination and by using proprietary algorithms (Intelligent Activity Classification) to classify activities into sitting, standing, or stepping. For the activPAL monitor, Basset et al. [20] postulated that once the angle of inclination exceeds approximately 20° from horizontal, the monitor predicts standing (i.e., upright position). In our laboratory experiments with the protractor, the angle of inclination representing standing was approximately 10–20° from horizontal for the transition from standing to sitting (Fig 2B). However, it is important to note that it was not the primary purpose of our study to determine the precise angle thresholds used by the ActiGraph and activPAL to determine different postures. Therefore, the measurements with the protractor must be interpreted with caution and should be considered rather exploratory, in addition to our measurements in the workplace.

In our study, postural transfer was detected with slightly higher accuracy by the ActiGraph monitor. One task, especially, that included short periods of changes in postures including sitting, standing, and squatting, was difficult to capture by the activPAL. This is supported by a previous study, in which the ActiGraph—compared with the activPAL—detected many more postural transitions (e.g., standing-to-walking, walking-to-standing, and sitting-to-walking) including stepping during a free-living protocol [11]. Differences in algorithmic hierarchy [11] and/or structure are likely the cause of the observed between-monitor differences in the detection accuracy of short postural changes.

Despite proper initialization by the investigator, the activPAL malfunctioned in one of 40 individual experiments (2.5%), coding the first 8 tasks incorrectly as standing. We decided to keep these individual measurements in the analysis, because they reflect an important aspect of measurement quality. In prior studies using the activPAL and ActiGraph simultaneously in the laboratory setting and under free-living conditions, equipment malfunction was reported for the activPAL, but not the ActiGraph [11, 15, 16]. Monitor malfunction for the activPAL was reported to be caused by the “future start” function during monitor initialization [15], and resulted in incorrect classification of posture during the entire measurement period [11].

It is important to note that our findings cannot be easily transferred to free-living conditions. In contrast to our controlled laboratory experiment, showing an overall high agreement between activPAL and ActiGraph for workplace-related tasks, between monitor accuracy to measure physical activity and sedentary behaviour under free-living conditions appears highly variable [21–23]. In this context, sensor placement is likely to have a major impact on between monitor agreement with studies showing excellent agreement when both devices were worn on the thigh [21], compared to other studies, in which the devices were worn around the wrist or hip (ActiGraph) and thigh (activPAL) [22, 23]. Additionally, our subjects were explicitly instructed to take a certain position (e.g., sitting on a chair with legs outstretched, standing in front of a whiteboard) and to remain in this position for a given time. During daily life activities, an individual’s movement behavior is possibly different as in our laboratory setting, and a greater variability in measurement precision could be expected for both devices.

### 4.1 Limitations

This study was designed to assess the criterion validity of the activPAL and ActiGraph to categorize a variety of different postures and motions in an experimental laboratory setting. While this study provides important insights into potential measurement problems and the misclassification bias of activity monitors for specific tasks in the workplace, our results are not generalizable to all office workplace situations and free-living conditions. Moreover, it is currently unclear to what extent misclassification of specific tasks affects an individual’s workplace sedentary behavior assessment during longer term measurements. Further, we have chosen a variety of different tasks to measure posture and motion at the workplace, but certain postures such as sitting on an ergonomic chair or seat ball were not covered in this study. Finally, we studied a group of healthy, normal weight individuals, our data may not be generalizable to people with obesity and/or joint disease who may be unable to perform some of the workplace-related tasks.

## 5. Conclusions

Our results indicate that the ActiGraph and activPAL are highly accurate in categorizing a variety of different postures and motions in the laboratory office setting. However, between-monitor differences were noted for some non-standard postures. The ActiGraph appears to be slightly more sensitive than the activPAL with respect to the measurement of sitting and postural transitions of short duration, whereas the activPAL seems to be slightly more accurate in capturing standing postures.

## Supporting information

S1 Appendix Experimental protocol: Instructions for study participants. (DOCX)

S2 Appendix Individual data from all participants. (XLSX)

S1 Table Evaluation criteria for the classification of sitting, standing, stepping, and postural transfer for the activPAL (AP) and ActiGraph (AG). (DOCX)

S2 Table Classification accuracy of the activPAL in detecting different workplace-related tasks in repeated measurements (N = 20 participants). (DOCX)

S3 Table Classification accuracy of the ActiGraph wGT3X-BT in detecting different workplace-related tasks in repeated measurements (N = 20 participants). (DOCX)
