# Supplementary material for: Criterion validity of the ActiGraph and activPAL in classifying posture and motion in office-based workers: A cross-sectional laboratory study
Source: PLoS One. 2021 Jun 2;16(6):e0252659. doi: 10.1371/journal.pone.0252659 (PMC8171934; doi:10.1371/journal.pone.0252659)
Supplement: S1 Appendix — (DOCX) [file pone.0252659.s001.docx]

**S1 Appendix. Experimental protocol: Instructions for study participants**

Please take a seat on the office chair and read these instructions. If needed, the height of the backrest can be adjusted. You can also adjust seat height and seat depth. Make sure that the resistance that you feel when leaning back (tilt tension) is right for you. The backrest should be securely fixed at the start of this exercise.

When you are seated, the height of the desk should be at about the level of your belly button. Please type out the text in bold that you see below. Typing mistakes are allowed. What is important is that you understand the content.

***Note down date: time: (hr min sec) age: sex: height: weight:***

***Here is a set of instructions; please follow the instructions. If anything is not clear, please ask the instructors.***

***It is important that your posture is relaxed, even when you are asked to assume a certain position/pose.***

***During this entire exercise, you will be observed and guided by the instructors. The instructors will be taking notes. Please do not be disturbed by this.***

***You will be asked several times to note down the time. The time should be written down in the following format: hr min sec. This is important so that the movement sequences can be evaluated correctly later on.***

***When writing down the time, please refer to the clock on the PC monitor. For tasks that are not performed on the PC monitor, the instructor will tell you the time.***

***Please write down the time now***

**TIME (category SITTING*):**

*The categories were added by the authors after the experiments were completed. The information in brackets was not visible for participants during the experiments.

Go to the window, open it, and sit down again. Check the clock and note down the time and remain seated for 30 seconds. Then go to the window, close it, and sit down again.

**TIME (category postural Transfer):**

Loosen the tilt tension of the backrest, note down the time, and then sit leaning all the way back for 2 minutes.

**TIME (category SITTING):**

Note down the time and then slowly seesaw with the backrest forwards and back for 2 minutes.

**TIME (category SITTING):**

Fix the backrest all the way forward, sit with your legs stretched out completely. Note down the time and remain like this for 2 minutes.

**TIME (category SITTING):**

Adjust the backrest all the way back again. Stretch out your legs completely. Then note down the time and remain like this for 2 minutes.

**TIME (category SITTING):**

Fix the backrest all the way forward again. Cross your right leg over your left knee. Then note down the time and remain like this for 2 minutes.

**TIME (category SITTING):**

Stand up and raise the desk height to about the height of your belly button. Stand still at the table. Then note down the time and remain like this for 2 minutes.

**TIME (category STANDING):**

Lower the desk back to sitting height and sit down. Note down the time and then jiggle your right foot as if you were nervously waiting for something. Try to do this for 2 minutes.

**TIME (category SITTING):**

Note down the time. Then go to the bookcase and get the clipboard/ballpoint pen from the bookcase. Squat down to get the clipboard/ballpoint pen. Place the objects on the desk, sit down, and note down the time again.

**TIME (category postural Transfer):** before getting the objects:

**TIME (category postural Transfer):** after getting the objects:

Now get the footrest, place it under the desk, and place both feet on it comfortably. Then note down the time and remain like this for 2 minutes.

**TIME (category SITTING):**

Imagine that you have to work at the computer for a long time (reading, entering text, surfing, …). Position yourself at the PC as you would under usual conditions. Then note down the time and remain like this for 2 minutes.

**TIME (category SITTING):**

Please save the document and then print it. Note down the time. Then take the clipboard/pen and go to the printer and read the further instructions on the printout.

**TIME (category STEPPING):**

Attach the printout to the clipboard and note down the time and then remain standing for 2 minutes.

**TIME (category STANDING):**

Note down the time. Then go to the bookcase and go into a squat. Then stand at the bookcase for 2 minutes.

**TIME (category postural Transfer):**

Go to the break room and sit down with your knees bent at right angles. Then note down the time and remain like this for 2 minutes.

**TIME (category SITTING):**

Sit down with your lower legs bent way back under the seat of the chair. Then note down the time and remain like this for 2 minutes.

**TIME (category SITTING):**

Go back to the desk with the printout, clipboard, and pen. Raise the desk again to the height of your belly button, note down the time, and then answer the questions while standing.

**TIME (category STANDING):**

- Do you use a standing desk or adjustable desk (at home or at work)?

- Have you already planned your next big vacation?

- In your free time, approximately how many hours do you work at the PC?

- When was the last time you went swimming?

Take the footrest and put it under the desk. Note down the time and then answer the following questions while standing. While standing, keep your right foot on the footrest the whole time.

**TIME (category STANDING):**

- On a work day in the office, how many hours do you work on average?

- Do you exercise regularly?

- Besides your office chair, what other things to sit on do you use regularly when working at the

desk/PC?

- Do you have pets?

Go to the standing desk and place your right foot on the lower shelf (your thigh will be at approximately a right angle to the hip). Then note down the time and remain like this for 2 minutes.

**TIME (category «SITTING»):**

Go to the floor below, taking the clipboard. Note down the time and then remain in front of the notice board for 2 minutes.

**TIME (category STANDING):**

Note down the time and then walk around the block for 2 minutes. Then go back to the office.

**TIME (category STEPPING):**

Imagine that you have to only read a longer text on the monitor. Seat yourself the way you normally would to read the text on the monitor. Then note down the time and remain like this for 2 minutes.

**TIME (category SITTING):**

Put your legs up on the desk. Then note down the time and remain like this for 2 minutes.

**TIME (category SITTING):**

**QUESTION:** Comments on activPAL or ActiGraph regarding wearing comfort, fit, and hold; how well this exercise covered your everyday office life (what was missing?):

**END**

**Thank you very much for your participation.**

## ***Experimental tasks*** *(point 2.4 in the original manuscript)*

The protocol consisted of 24 pre-defined tasks representing sitting (N = 11), standing (N = 5), stepping (N = 5), and postural transitions (N = 3) around the workplace, with each task lasting a maximum of 2 minutes. In brief, sitting was tested in different positions, for example with the legs stretched out or placed on a footrest. Several standing tasks were tested, such as reading from the computer screen, standing at the pin board while reading a newsletter, waiting for a printout at the printer, and standing with one foot on the footrest with slightly bent knees. Stepping was assessed while ascending/descending stairs and walking on flat ground around the institutional building. Postural transitions included transfer from standing to squatting, or standing up from and sitting down on a chair, with a few steps in-between to open and close the window.

For the tasks on the computer screen, the participants had to work through the experimental protocol themselves, with instructions being provided in a Word document (Microsoft Word 2016). The participants had to note the time from the computer clock at the beginning of each task in the Word document. The observer instructed the participants on all tasks that were not performed at the computer workstation. The detailed experimental protocol including the description of the different tasks can be found elsewhere (see supplementary material 1).

Furthermore, we examined one special position: standing with the right foot placed on a bookcase with an approximately 90º hip angle; this served as a positive control, since we expected both devices to categorize the position as sitting.

At the end of the experiments, the participants were asked about positions that the investigators missed, in order to address differences between the experimental protocol and their personal habits, and regarding the wearing comfort of the two activity monitors.
