# Supplementary material for: Criterion validity of the ActiGraph and activPAL in classifying posture and motion in office-based workers: A cross-sectional laboratory study
Source: PLoS One. 2021 Jun 2;16(6):e0252659. doi: 10.1371/journal.pone.0252659 (PMC8171934; doi:10.1371/journal.pone.0252659)
Supplement: S1 Table — (DOCX) [file pone.0252659.s003.docx]

**S1 Table.** Evaluation criteria for the classification of sitting, standing, stepping and postural transfer for activPAL (AP) and ActiGraph (AG).

| **Assigned Task** | **Expected code from monitor** | **Evaluation criteria** |
| --- | --- | --- |
| Sitting | AG: Sitting/Lying | At least six 15s-epochs coded as “sitting/lying” (AG) or “sedentary” (AP) for 2 min tasks (75%) or 80% of data coded as “sitting/lying” (AG) or “sedentary” (AP), if the assigned tasks lasted less than 2 minutes. |
|  | AP: Sedentary |  |
| Standing | AG: Standing | At least six 15s-epochs coded as “standing” (AG) or “upright” (AP) for 2 min tasks (75%) or 80% of data coded as “standing” (AG) or “upright” (AP), if the assigned tasks lasted less than 2 minutes. |
|  | AP: Upright (excluding Stepping) |  |
| Stepping | AG: Stepping | At least six 15s-epochs coded as “stepping” (AG) or “stepping” plus upright for (AP) for tasks lasting 2 minutes (75%). |
|  | AP: Stepping plus upright |  |
| Postural transfer  Example: Stand – Step – Squat – Stand – Step | AG: Standing – Stepping – Sitting/Lying – Standing  AP: Upright – Stepping – Sedentary – Upright | At least a short interruption of coded activity, i.e., change from “standing” (AG) or “upright” (AP) to “sitting/lying” (AG) or “sedentary” (AP). |
