# Supplementary material for: Criterion validity of the ActiGraph and activPAL in classifying posture and motion in office-based workers: A cross-sectional laboratory study
Source: PLoS One. 2021 Jun 2;16(6):e0252659. doi: 10.1371/journal.pone.0252659 (PMC8171934; doi:10.1371/journal.pone.0252659)
Supplement: S2 Table — (DOCX) [file pone.0252659.s004.docx]

**S2 Table.** Classification accuracy for the activPAL to detect different workplace-related tasks during repeated measurements (n=20 participants).

|  | **activPAL^TM^ micro** | | | | |
| --- | --- | --- | --- | --- | --- |
| **ASSIGNED TASKS** | 1^st^ *and* 2^nd^ task correctly classified (N) | Only 1^st^ task correctly classified (N) | Only 2^nd^ task correctly classified (N) | 1^st^ *and* 2^nd^ task not correctly classified (N) | *p*-value |
| **SITTING (n=11)** |  |  |  |  | 0.383 |
| Typing text on keyboard | 19* | 0 | 1 | 0 | 0.317 |
| Feet placed on footstool | 20 | 0 | 0 | 0 | 1.000 |
| Moving backrest forward and backward | 19* | 0 | 1 | 0 | 0.317 |
| Fidgeting with the right leg | 19* | 0 | 1 | 0 | 0.317 |
| Right leg crossed at the left knee | 20 | 0 | 0 | 0 | 1.000 |
| Feet placed on the desk | 20 | 0 | 0 | 0 | 1.000 |
| Backrest fixed fully backward | 19* | 0 | 1 | 0 | 0.317 |
| Legs outstretched | 6 | 2 | 3 | 9 | 0.655 |
| Legs outstretched & backrest fully backward | 15 | 3 | 1 | 1 | 0.317 |
| On a chair with both feet on the ground | 18 | 2 | 0 | 0 | 0.157 |
| On a chair with lower legs bent far back under the seat of the chair | 11 | 1 | 5 | 3 | 0.103 |
| **STANDING (n=5)** |  |  | |  | 1.000 |
| In front of the pin board | 20 | 0 | 0 | 0 | 1.000 |
| Desk adjusted for standing position | 20 | 0 | 0 | 0 | 1.000 |
| Desk adjusted for standing position & typing text on keyboard | 20 | 0 | 0 | 0 | 1.000 |
| Waiting for printout in front of the printer | 20 | 0 | 0 | 0 | 1.000 |
| Desk adjusted for standing position & right foot placed on footrest | 20 | 0 | 0 | 0 | 1.000 |
| **STEPPING (n=5)** |  |  |  |  | 1.000 |
| Walking outside around the building | 20 | 0 | 0 | 0 | 1.000 |
| Stairs, ascending (two flights) | 20 | 0 | 0 | 0 | 1.000 |
| Stairs, ascending (one flight) | 20 | 0 | 0 | 0 | 1.000 |
| Stairs, descending (three flights) | 20 | 0 | 0 | 0 | 1.000 |
| Stairs, descending (one flight) | 20 | 0 | 0 | 0 | 1.000 |
| **POSTURAL TRANSFER (n=3)** |  |  |  |  | 0.289 |
| Getting up from and sitting down on a chair to open and close the window *(Sit–Step–Stand–Step–Sit–Step–Stand–Step–Sit)* | 18 | 0 | 2 | 0 | 0.157 |
| Getting up from a chair and walking a few meters to take a clipboard from the bottom of a bookcase, returning to the chair and sitting down again *(Sit–Step–Squat–Step–Sit)* | 19* | 0 | 1 | 0 | 0.317 |
| Standing in front of the printer, taking the printout, walking a few meters and squatting down to place the printout in the lower part of the bookcase *(Stand–Step–Squat–Stand–Step)* | 7 | 5 | 1 | 7 | 0.103 |
| **SPECIAL POSITION (n=1)** |  |  |  |  |  |
| Standing with the right foot placed on the bookcase with ~90° hip angle | 18 | 1 | 1 | 0 | 0.317 |

Number of correctly classified tasks for the activPAL monitor based on repeated individual measurements (N=40) from 20 study participants. The *p*-values denote differences in the classification accuracy to detect the pre-specified tasks during repeated measurements. * During one individual experiment, the activPAL monitor malfunctioned, coding the first tasks constantly as standing.
